# Improved Structure and Function in Autosomal Recessive Polycystic Rat Kidneys with Renal Tubular Cell Therapy

**DOI:** 10.1371/journal.pone.0131677

**Published:** 2015-07-02

**Authors:** K. J. Kelly, Jizhong Zhang, Ling Han, Malgorzata Kamocka, Caroline Miller, Vincent H. Gattone, Jesus H. Dominguez

**Affiliations:** 1 Department of Medicine, Indiana University School of Medicine, Indianapolis, IN, United States of America; 2 Department of Anatomy, Indiana University School of Medicine, Indianapolis, IN, United States of America; 3 Department of Medicine, Veterans Affairs Medical Center, Indianapolis IN, United States of America; Center for Molecular Biotechnology, ITALY

## Abstract

Autosomal recessive polycystic kidney disease is a truly catastrophic monogenetic disease, causing death and end stage renal disease in neonates and children. Using PCK female rats, an orthologous model of autosomal recessive polycystic kidney disease harboring mutant *Pkhd1*, we tested the hypothesis that intravenous renal cell transplantation with normal Sprague Dawley male kidney cells would improve the polycystic kidney disease phenotype. Cytotherapy with renal cells expressing wild type *Pkhd1* and tubulogenic serum amyloid A1 had powerful and sustained beneficial effects on renal function and structure in the polycystic kidney disease model. Donor cell engraftment and both mutant and wild type *Pkhd1* were found in treated but not control PCK kidneys 15 weeks after the final cell infusion. To examine the mechanisms of global protection with a small number of transplanted cells, we tested the hypothesis that exosomes derived from normal Sprague Dawley cells can limit the cystic phenotype of PCK recipient cells. We found that renal exosomes originating from normal Sprague Dawley cells carried and transferred wild type *Pkhd1* mRNA to PCK cells *in vivo* and *in vitro* and restricted cyst formation by cultured PCK cells. The results indicate that transplantation with renal cells containing wild type *Pkhd1 *improves renal structure and function in autosomal recessive polycystic kidney disease and may provide an intra-renal supply of normal *Pkhd1 *mRNA.

## Introduction

Most patients with autosomal recessive polycystic kidney disease (ARPKD) who survive the neonatal period suffer from severe renal complications early in childhood [1,2]. ARPKD is a monogenetic disease resulting from mutations in *PKHD1*, which encodes the cilial protein fibrocystin [3]. Polycystic kidney disease (PKD) is currently incurable, although several approaches have shown benefit [2,4–13]. The definitive treatment would be genetic, but, there are no current safe and effective clinical modes of gene transfer [14–16]. Hence, we examined a novel therapeutic strategy employing adult kidney cell transplantation, previously successful in other renal failure models [17–20]. Our earlier data include successful long-term kidney cell engraftment and improved renal structure and function in experimental diabetic nephropathy [19], as well as following cell auto-transplants in a chronic kidney disease model [20]. In the present study, we used adult primary kidney cells reprogrammed (via a non-viral vector) to express the tubulogenic protein serum amyloid A1 (SAA,[17]).

We now report the results of intravenous renal cell transplantation (IRCT) in the PCK rat, an orthologous model of ARPKD, derived from Sprague Dawley (SD) rats [21]. The overall aim was improved structure and function and we believe that goal was achieved. The transplanted renal cells from SD rats expressed normal *Pkhd1* and improved renal structure and function in the PCK rats (as compared to rats that received no cells). Engrafted donor cells were identified in recipient kidneys 15 weeks after the last cell dose. We also report that SD renal exosomes carry and transfer wild type *Pkhd1* exo-mRNA and can limit cyst formation in matrigel matrices. We propose that IRCT is a safe and very effective means to deliver the wild type *Pkhd1* gene and, more importantly, prevent progressive CKD in PKD. IRCT has the advantage that renal cells from one normal rat are sufficient to transplant multiple diseased animals, administration is non-invasive and the side effects of immunosuppression are obviated.

## Materials and Methods

### Primary Renal Tubular Cells

Primary renal cells from one age-matched male SD rat (Harlan, Indianapolis, IN) were equally distributed to four PCK female rats, one in each of the four cell treatment groups described below. After insuring adequate anesthesia, both kidneys were removed, the cortices minced in S1 medium (Ham’s F-12/DMEM) with type 4 collagenase (Worthington, Lakewood, NJ), 6 mg/dl, at 37°C in 38% O2 and 5% CO2 for 50 minutes. Renal tubules were then separated by percoll gradient [20], divided into two sets, and transfected by electroporation. Control (SAA negative) tubules were co-transfected with empty vector pcDNA3.1 (30 ug), pAcGFP1-C1 (15 ug, GFP is the cytosolic label used to track cells in vivo, Clontech, Mountain View, CA). For SAA+ cells, pcDNA3.1 was replaced with pcDNA3.1-SAA1 plasmid, 30 ug, manufactured and sequenced in our laboratory as previously reported [17,20]. The isolated tubules were a mix of different tubule segments: approximately 1/4 proximal (positive for organic anion transporter 1), 20% thick ascending limb (positive for Tamm Horsfall protein), 15% collecting tubule (positive for aquaporin-2) and 5% distal convoluted tubule (positive for thiazide-sensitive co-transporter) [20]. Transfection efficiencies were >70% [20].

The co-transfected tubules were cultured in S1 medium with hepatocyte growth factor, 200 ng/ml; epidermal growth factor, 400 ng/ml (R&D Systems, Minneapolis, MN); hydrocortisone 100 ug/ml; insulin, 35 ug/ml; transferrin, 32 ug/ml; sodium selenite 42 ng/ml (Sigma, St. Louis MO); and 20% fetal calf serum. G418, 75 ug/ml, was added after 48 hours of culture for selection. In preparation for transplantation, male renal tubular cells were lightly trypsinized after 7–8 days in culture, washed in PBS, and 10^6^ cells injected intravenously in the tail vein of PCK female rats at 6 (2 days after surgery, below), 8 and 10 weeks of age.

### Animal Protocols

#### Ethics Statement

This study was carried out in strict accordance with the recommendations in the Guide for the Care and Use of Laboratory Animals of the National Institutes of Health. The protocol was approved by the Institutional Animal Care and Use Committee of the Indiana University School of Medicine (permit 3616). All surgery was performed under isofluorane anesthesia and all efforts were made to minimize suffering. This included the administration of analgesia (buprenorphine) postoperatively. Although criteria (including minimal movement, not taking food, loss of more than 15% of body weight) for early euthanasia were in place, early euthanasia was not necessary. The animals were monitored regularly: continuously while under anesthesia and then daily. The method of euthanasia, overdose of a barbituric acid derivative with subsequent exsanguination, is consistent with the American Veterinary Medical Association guidelines for the Euthanasia of Animals.

#### Experimental Design

Female PCK rats (Charles River, Wilmington, MA) were assigned to the 2 control and 4 experimental groups in a blinded manner. Rats underwent left renal ischemia or sham surgery at 6 weeks of age (between approximately 9 and 11 am) in laboratory space designated for rodent surgery. Anesthesia was accomplished with inhaled isofluorane (0.5–1% to effect) prior to occlusion of the left renal pedicle for 50 minutes. Sham surgery consisted of an identical procedure except the renal pedicle was not clamped [22]. Anesthesia was chosen to minimize recovery time and alterations in blood pressure that might cause further renal injury. The rats were infused at 6, 8 and 10 weeks of age with donor cells (10^6^ cells/infusion): one control (sham) group and one postischemia group received control (SAA-) cells. Additional control (sham) and postischemia groups received SAA+ cells. Control sham and postischemia rats received no cells. Weights, sera and urine were collected biweekly and chemistries were measured by the Indianapolis VA clinical laboratory. Urine protein was measured via ELISA according to the manufacturer’s protocol (Exocell, Philadelphia, PA). Cystic change was quantified using point count stereology as described [23]. Dynamic contrast computed tomography was performed on anesthetized animals 1–2 weeks before sacrifice using a high speed CT scanner as described [24]. To examine the role of exosomes in the transfer of wild type *Pkhd1*, one additional group of PCK rats underwent anesthesia as above with injection of exosomes (20ug protein, exosomes from approximately 2 x 10^6^ cells) into the renal pelvis and sacrificed 24 hours later. Additional supporting information describing more details of animal use is in supplementary information (S1 File).

### Histology and immunohistochemistry

Kidney sections were fixed in 3.8% paraformaldehyde, paraffin embedded and 5 μM sections obtained for Masson’s trichrome to stain collagen blue and periodic acid Schiff (PAS) to evaluate morphology. The areas of glomerular and peritubular fibrosis were quantified in blinded sections and expressed as fractional areas, covering all available sections. Additional kidney sections were immunostained with anti-CD31 (PECAM) antibody (Santa Cruz Biotechnology, Santa Cruz, CA) and Texas-Red conjugated secondary antibody (Jackson Immunoresearch, West Grove, PA), to visualize the microvasculature. Kidney sections were also stained with anti-pan-keratin and anti-vimentin (both Cell Signaling, Danvers, MA) and Texas-Red conjugated secondary antibodies. Anti-organic anion transporter 1 (OAT1, Alpha Diagnostics International, San Antonio, TX), anti-Tamm Horsfall protein (THP, Millipore, Temecula, CA), anti-aquaporin-2 (AQP, Millipore) and anti-thiazide-senstiive co-transporter (TSC, Alpha Diagnostics International) were used in combination with Texas Red conjugated secondary antibodies as specific tubule segment markers. Paraformaldehyde fixed 100 μm kidney sections (Vibratome, St. Louis, MO) were immunostained with rabbit primary anti-SAA antibody [18,20] and Texas Red conjugated secondary antibody. Nuclei were labeled with DAPI (Molecular Probes, Eugene, OR). Fluorescence images of immunostaining and expressed GFP were collected with a Leica DMI 3000B fluorescence microscope. Confocal images were obtained with an Olympus FV1000-MPE microscope. Quantification of immunostaining was performed using Metamorph software in blinded sections.

### Fluorescent *in situ* hybridization (FISH) of the Y chromosome

FISH was used to localize the Y chromosome in female kidneys 15 weeks after IRCT with male renal cells as previously reported [20] employing the fluorescent labeled rat Y chromosome probe (Rat Idetet Chr Y Paint probe red, ID556, ID Labs Biotechnology Inc. London ON, Canada). Sections were counterstained with DAPI prior to imaging with Leica DMI 3000B fluorescence microscope.

### Renal SAA1 mRNA

The murine SAA1 mRNA was amplified from renal RNA (isolated using Trizol, Invitrogen, Grand Island, NY via the supplier’s protocol) using PCR System 2400 (Perkin Elmer, San Jose, CA) with the following primers [17]:

Forward 1: 5’-CGCCACCATGGAGGGTTTTTTTCATTTGTTCAC-3’

Forward 2: 5’-TACAGGCTAGCGCCACCATGGAGGGTTT-3’

Reverse 1/2: 5’TCAGGTGGATCCCTCAGTATTTGTCAG-3’

### Identification of DNA encoding the male sex-determining region on chromosome Y (SRY*)* in female kidneys [19]

DNA was extracted from the recipient kidneys with the Wizard Genomic DNA Purification Kit as indicated by the manufacturer (Promega, Madison, WI). The specific SRY DNA was then amplified from extracted kidney DNA using PCR System 2400 with the following primers [25]:

Forward: 5’-AAGCGCCCCATGAATGC-3’

Reverse: 5’-AGCCAACTTGCGCCTCTCT-3’

### Genotyping

Identification of wild type and mutated *Pkhd1* genes in the PCK rats was performed via PCR (as above) using the primers specified by Charles River:

Mut-Forward: 5’-AAG CCA AAT CTT TCT CTT TTC CT-3’

Mut-Reverse: 5’- CTT GCT GTC CGA ATA CCA C -3’

Wild type-Forward: 5’-ACT GCC TTT TAC TGA AGC ATT TAA C-3’

Wild type-Reverse: 5’- TGG AAG GAA AAG TTG CCC T -3’

### Exosome studies

Primary renal tubule cells from normal Sprague Dawley rats (Harlan, Indianapolis, IN) were isolated as above. After 2 days in culture, S1 medium with exosome-free fetal calf serum was used. Two days later, the cell culture supernatant was centrifuged at 300g for 10 minutes to remove cells, 2000g x 10 minutes to remove dead cells, 10,000g x 30 minutes to remove cells debris. The resultant supernatant was centrifuged at 100,000g x 70 minutes, washed and centrifuged again at 100,000g x 70 minutes to obtain exosomes. After fixation in 2% paraformaldehyde/2% glutaraldehyde/0.1M phosphate buffer, the sample was adsorbed to a 200–400 mesh carbon/formvar coated grid and the negative stain (Nanovan, Nanoprobes, Yaphank, NY) added. Exosome isolation was then verified by electron microscopy (Tecnai G2 12 Bio Twin microscope [FEI, Hillsboro, OR] equipped with an AMT CCD camera [Advanced Microscopy Techniques, Danvers, MA]). Prior to their addition to cultured PCK cells, SD exosome RNA was labeled with red fluorescent dye and exosome protein with green fluorescent dye via Exo-Glow (SBI, Mountain View, CA) according to the supplier’s protocol. PCK tubular cells were isolated by collagenase digestion and cultured as for Sprague Dawley cells (above). When the cells were 50–70% confluent, the medium was changed to S1 medium with 10% exosome free fetal calf serum and fluorescently labeled exosomes (10μg protein/10^6^ cells) added to the cells and imaging performed approximately 16 hours later. Uptake of exosomes was documented by PCR genotyping (above). For these studies, prior to incubation with exosomes, some PCK cells were treated with cytochalasin D and chloropromazine (each 10ug/ml) to block exosome uptake (actin polymerization and endocytosis, respectively). In separate studies, exosome treated cells were cultured for 2 days prior to resuspension in matrigel (BD Biosciences, Bedford, MA) at a concentration of 100,000 cells/ml and incubated in glass bottom dishes. In some studies, PCK and SD cells were cultured together in the following proportions (Table 1)

**Table 1:** 

PCK	100%	98%	90%	80%	0%
SD	0%	2%	10%	20%	100%

Cyst number was quantified in blinded images at 4 days. At 7 days, the cells were fixed in 4% paraformaldehyde, permeabilized in 0.1% Triton and incubated with rhodamine phalloidin (1:200) and Hoescht 3342 (both Life Technologies, Carlsbad, CA). Two photon images were acquired with an Olympus Fluoview FV-1000 MPE system (Olympus America, Central Valley, PA) using 839 nm excitation wavelength and an Olympus XLPLN 25x, NA 1.05 water immersion objective. Z-stacks were collected to evaluate cyst formation in 3D. Volume rendering was performed using Amira software (FEI, Burlington, MA).

### Immunoblotting

Exosome and cell lystate samples and fibrocystin protein control (Santa Cruz Biotechnology, Santa Cruz, CA) were fractionated by electrophoresis through 16.5% polyacrylamide Tris-tricine gels. After transfer and blocking, blots were incubated with anti-fibrocystin or anti-CD63 (Santa Cruz).

### Statistics

The experimental unit was one culture dish or one kidney (as right and left kidneys were treated differently). For albuminuria and BUN, the experimental unit was a single animal. Data are expressed as means ± 1 standard error. Analysis of variance was used to determine if differences among mean values reached statistical significance. Tukey’s test was used to correct for multiple comparisons. Student's t test (2 tailed, 2 sample, unequal variance) was used for comparisons between groups (GraphPad Prism, LaJolla, CA). The null hypothesis was rejected at p<0.05.

## Results

Female PCK rats received either no cells, control cells or SAA+ cells intravenously when 6, 8 and 10 weeks old and were sacrificed at 25 weeks of age. Renal tubular cells from normal male SD rats were either transfected with empty vector (control cells) or SAA1. Both control and SAA+ cells were also transfected with green fluorescent protein (GFP) for tracking purposes.

**Fig 1:**
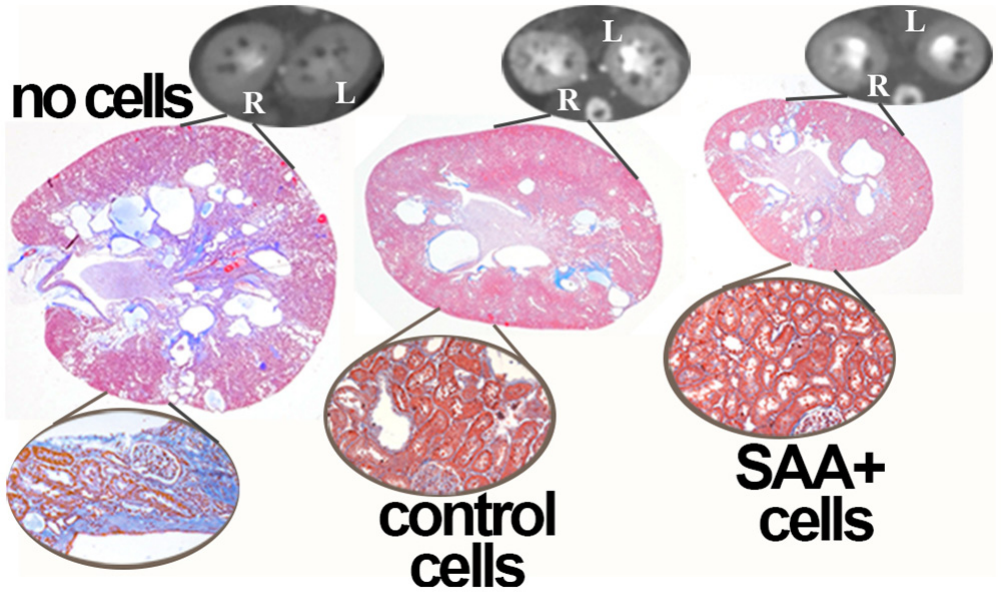
Cytotherapy decreases cyst burden. When compared to no cell transplant groups, treatment of PCK rats wiSAA+ or control cells improves cyst volume and structure at 25 weeks of age. The termination point was 15 weeks after the final cell transplant. Representative dynamic contrast CT images and PAS stained and trichrome stained sections (insets) are presented.

**Fig 2:**
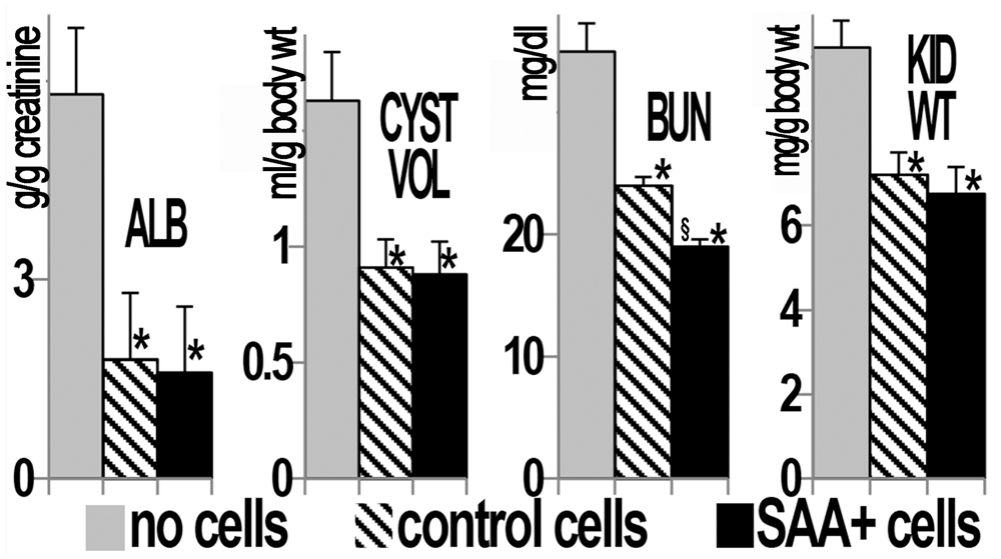
Improvement in structure and function in PCK rat kidneys with cell transplant. When compared to no cell transplant groups, treatment of PCK rats with SAA+ or control (A) cells improves albuminuria (ALB), total cyst volume (CYST VOL), blood urea nitrogen (BUN), and kidney weight (KID WT) at 25 weeks of age, 15 weeks after the final cell transplant. Albuminuria is presented as g/g creatinine, cyst volume as ml/g body weight, BUN as mg/dl, and kidney weight as mg/g body weight. *p<0.05 vs no cell group, §p<0.05 vs control cells

In Fig 1 is shown the marked improvement in cyst burden and renal histology in PCK rats that were transplanted with control A renal cells (containing wild type *Pkhd1*) and an even greater positive effect in groups that received B renal cells (containing both wild type *Pkhd1* and SAA1) when compared to PCK rats that did not receive cells. In addition to decreased total cyst volume and kidney weights, better renal function was observed in the cell transplant groups compared to the “no cell” rats as shown by decreased albuminuria and serum blood urea nitrogen (BUN) (Fig 2). Mean BUN was lower in the group that received SAA+ cells than in the control (SAA-) cell group.

**Fig 3:**
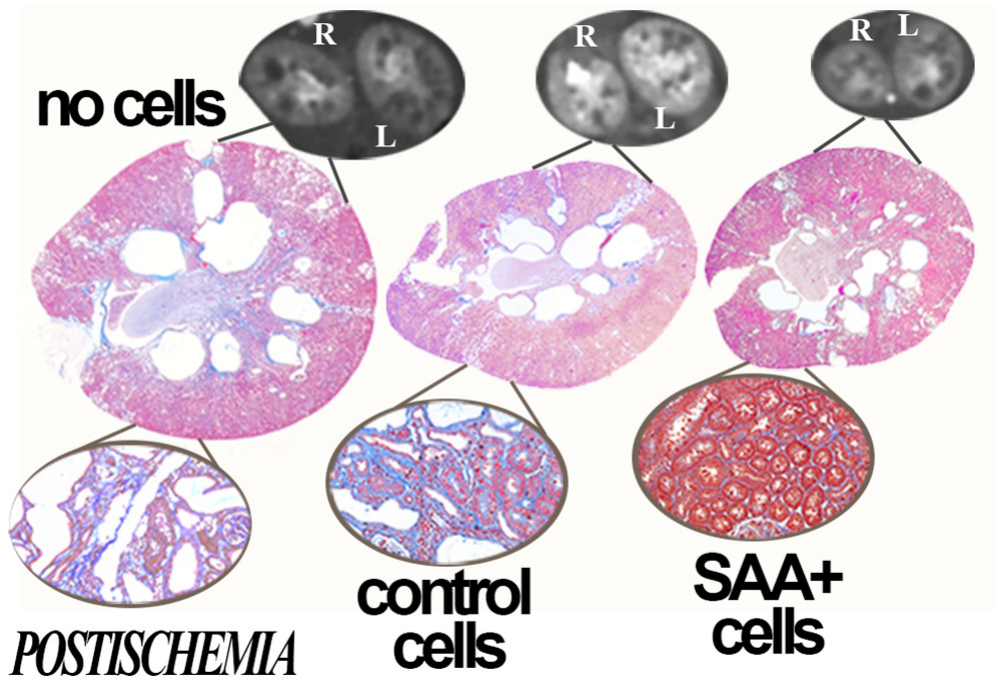
Protection in postischemia kidneys. When compared to no cell transplant groups, treatment of PCK rats with SAA+ or control cells also improves cyst volume and structure in postischemia kidneys at 25 weeks of age, 15 weeks after the final cell infusion. Representative dynamic contrast CT images and PAS stained and trichrome stained sections (insets) are presented.

**Fig 4:**
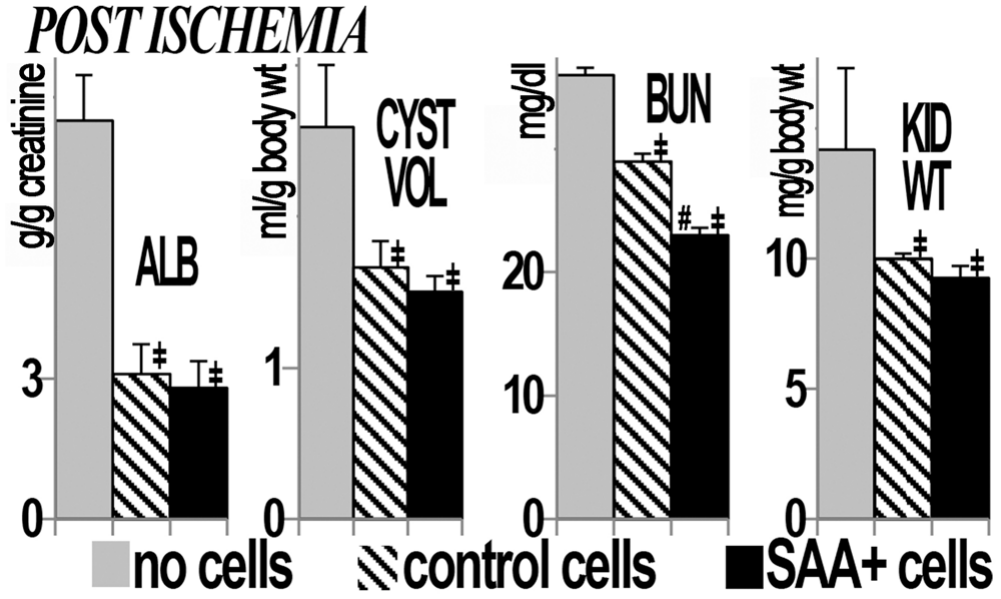
Improvement in structure and function in postischemia PCK rat kidneys with cell transplant. Treatment with SAA+ or control (A) cells improves albuminuria (ALB), total cyst volume (CYST VOL), blood urea nitrogen (BUN), and kidney weight (KID WT) in postischemia PCK rats. Albuminuria is presented as g/g creatinine, cyst volume as ml/kidney/g body weight, BUN as mg/dl, and kidney weight as mg/g body weight. ‡p<0.05 vs no cell/ischemia group, #p<0.05 vs control cell/ischemia group

**Fig 5:**
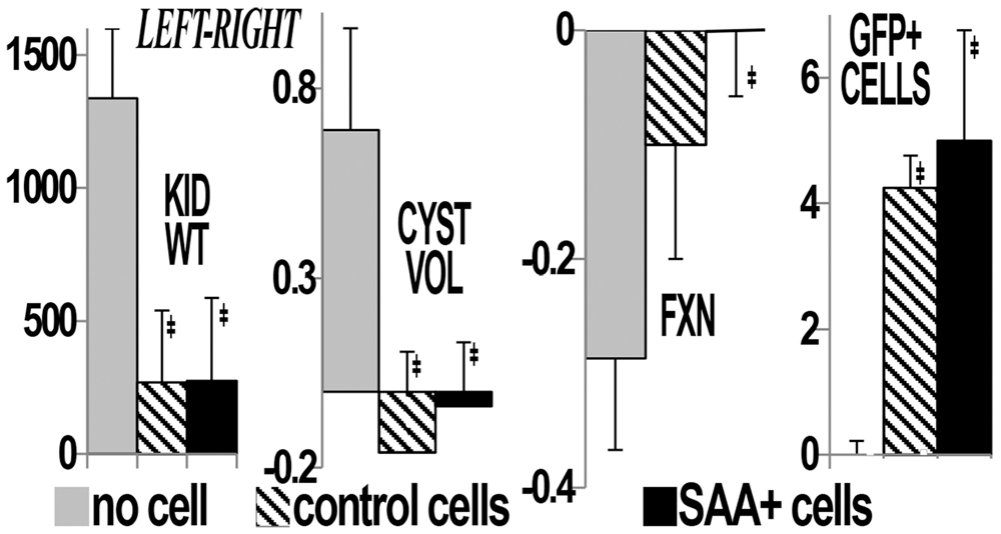
The effect of ischemia. Comparison of left (postischemic) and right (contralateral, sham) kidneys in postischemia groups shows that the difference in kidney weight, total cyst volume, split renal function by dynamic contrast computed tomography between left and right kidneys is attenuated in the groups that received cell transplantation. In addition, more GFP+ cells were found in the postischemic (vs sham) kidneys. ‡p<0.05 vs no cell/ischemia group.

We hypothesized that renal ischemia, common in PKD, would reduce the number of mutant cells through mutant cell death and facilitate engraftment of transplanted cells on the denuded basement membrane. Thus, an additional three PCK groups were subjected to left (unilateral) renal ischemia at 6 weeks of age and transplanted with either no cells, control cells or SAA+ cells. One of the control ischemia rats died when 23 weeks of age. In the postischemia groups, decreased total cyst volume, kidney weights, albuminuria and BUN were also observed in the rats that received cytotherapy (Figs 3 and 4). In addition, comparisons between the postischemic left and sham right kidneys showed improvement in kidney weight, total cyst volume, split renal function by dynamic contrast computed tomography and increased numbers of GFP+ cells/hpf in the postischemic kidney (Fig 5). Despite this, function and structure were more impaired in the postischemia groups. Interestingly, mean heart/body weight was lower in the cytotherapy groups (4.46, 3.66 and 3.69 mg/g body weight in no cell, control and SAA+ cell groups, respectively, p<0.01 vs no cell). There were no significant differences in animal weights (mean 302 ± 2.6 g) or final liver weights during the course of the study.

**Fig 6:**
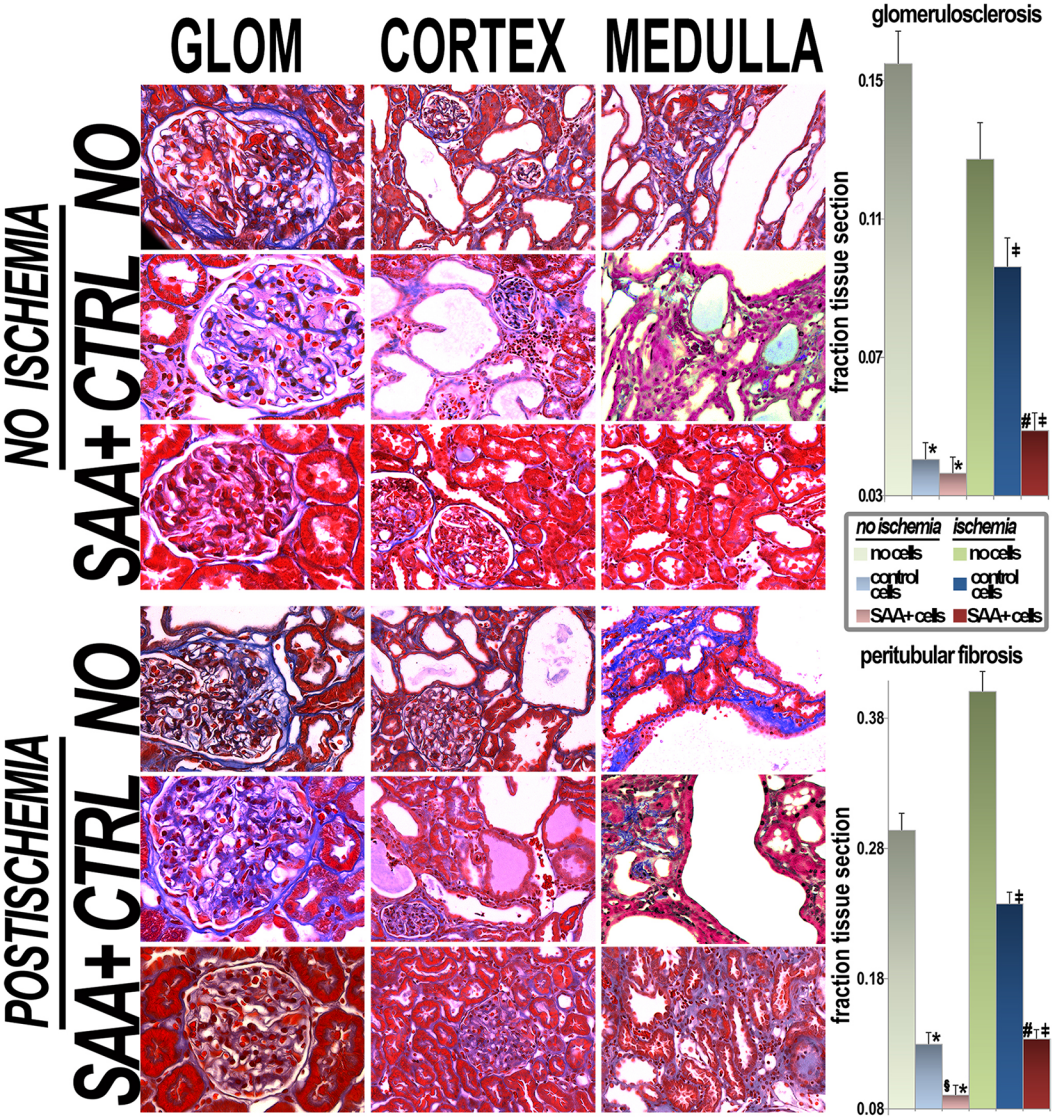
Histology. Representative trichrome stained sections (resulting in blue labeling of fibrous tissue) of glomeruli (glom), cortex and medulla in each of the 6 groups are presented. Quantification of glomerulosclerosis (in a total of 5602 glomeruli) and peritubular fibrosis (in 4249 microscope fields) is presented in the graphs. GLOM, glomeruli; NO, no cells; CTRL, control; *p<0.05 vs no cell group; ‡p<0.05 vs no cell/ischemia group; §p<0.05 vs control cell group; #p<0.05 control cell/ischemia group

Fibrosis is a significant contributor to decreased function in PKD [26]. In addition to improvements in function and total cyst volume, decreases in both peritubular fibrosis and glomerulosclerosis were also observed in treated kidneys. SAA+ cells results in larger improvements in fibrosis than SAA- cells (Fig 6).

**Fig 7:**
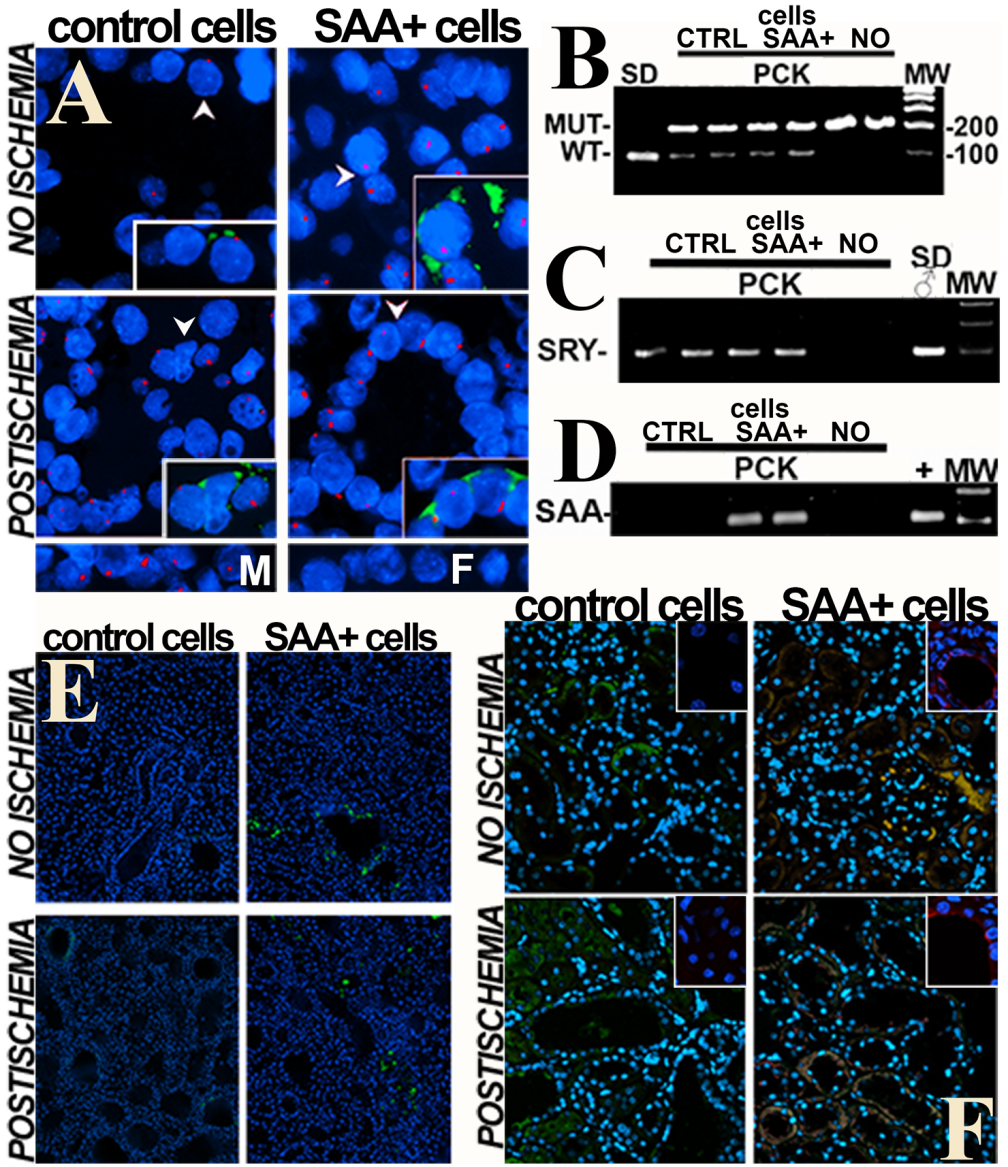
Engraftment of transplanted cells. Multiple methods were employed to demonstrate the persistence of infused cells in transplanted kidneys. (A) Fluorescence in situ hybridization (FISH) showed Y chromosome + (red) nuclei in kidneys of female rats that received male cells. The insets show green fluorescence of areas at arrows, demonstrating GFP and the Y chromosome in the same cells. Nuclei in this panel and panels E and F are stained with DAPI (blue). (B) Genotyping by PCR demonstrated the presence of both wild type (WT) and mutated (MUT) *Pkhd1* transcripts in kidneys from PCK rats that received SAA+ or control cells but not in PCK rats that received no cells. Only the wild type transcript is present in normal Sprague Dawley (SD) rats. (C) PCR for the SRY male determining gene showed similar results: SRY was present in female PCK rats transplanted with male cells and male (♂) SD but not female rats that received no cells. (D) mRNA for SAA is present only in groups that received SAA+ cells demonstrating transcriptional activity of the SAA gene from donor SAA+ cells. pcDNA3.3-SAA1 was used as the positive (+) control for SAA PCR. (E) GFP positive cells are also found in transplanted kidneys. (F) Immunostaining for SAA demonstrates co-localization (orange) with GFP in SAA+ groups. The insets in F show higher power confocal images for SAA in cyst epithelium. M = normal male; F = normal female; MW = molecular weight markers.

In contrast to stem cell transplant protocols, we have clearly documented engraftment of donor cells months after transplantation in other renal failure models [18–20]. Multiple independent techniques were used to verify this critical mechanistic point (Fig 7): (1) fluorescence in situ hybridization (FISH) showed the Y chromosome in female recipient kidneys transplanted with male cells, but not in normal females; (2) PCR genotyping demonstrated both mutated and wild type *Pkhd1* in the kidneys of transplanted rats, but not in those that did not receive cells; PCR detected both (3) DNA encoding the male determining SRY gene in female kidneys transplanted with male cells but not in control females and (4) SAA mRNA in kidneys that received SAA+ but not control (SAA-) cells or in rats not given cells; (5) fluorescence microscopy showed GFP+ cells in kidneys of rats that received GFP+ control or SAA+ cells and (6) co-localization of immunoreactive SAA with GFP in kidneys from rats that received SAA+ cells. In contrast to the kidneys, GFP+ donor cells were very rarely (< 1 cell/hpf) seen in lungs, spleen or liver in any of the groups. The majority of GFP+ cells were tubular with rare renal interstitial GFP+ cells (Fig 7). In summary, multiple independent tests showed that cell transplantation can deliver normal genes to cystic kidneys, which is the goal of “gene therapy.”

**Fig 8:**
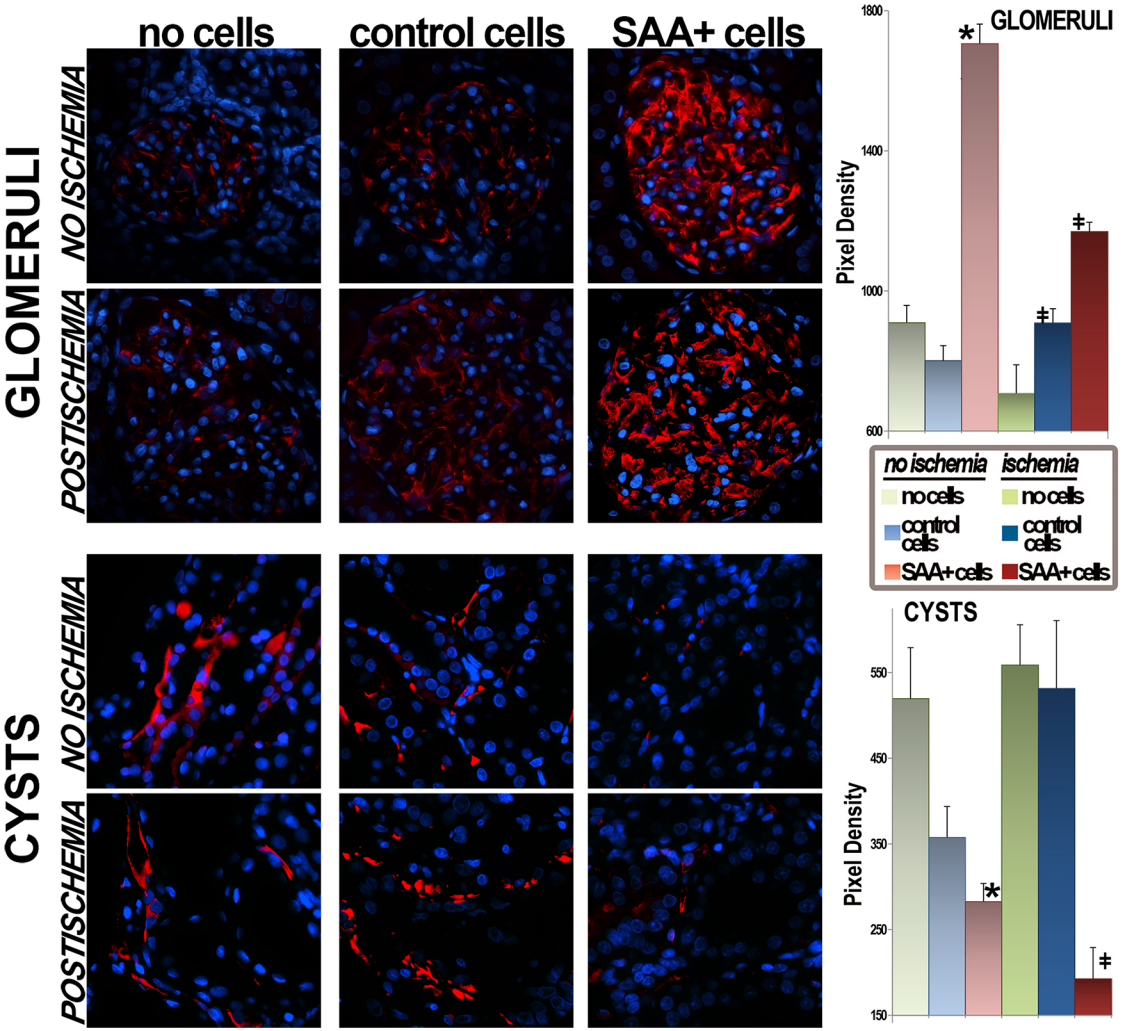
Protection of microvasculature with cytotherapy. Representative images stained for CD31 (platelet endothelial cell adhesion molecule [PECAM], red) show better preserved glomerular vasculature in SAA+ cell groups. In addition, less vasculature surrounding abnormal cystic epithelium is seen in the groups that received SAA+ cells. Quantification of red pixel density representing CD31 staining in 266 images is shown in the graphs. *p<0.05 vs no cell group; ‡p<0.05 vs no cell/ischemia group

We have postulated that the broad benefit seen with cell transplants [18–20] points to a general action, potentially explained by improved vasculature with better delivery of oxygen and nutrients. It is known that major renal microvascular abnormalities aggravate human PKD, promoting renal dysfunction and cyst enlargement [27]. Thus, the renal microvasculature was labeled with an anti-CD31 antibody to evaluate the role of cell transplantation, Fig 8. Representative images illustrate severe glomerular microvascular attenuation in control PCK rats and in those transplanted with SAA- cells. In contrast, glomerular vessels were much better preserved in the groups that received SAA+ cells. Pericystic hypervascularity, thought to contribute to cyst growth in human PKD [28] was markedly attenuated in cell treated rats.

**Fig 9:**
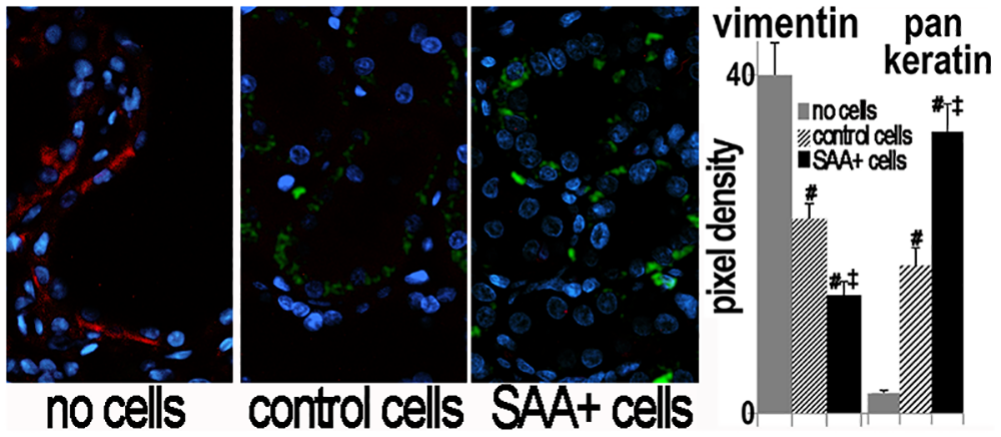
Decreased vimentin in kidneys after cell treatment. Vimentin expression, consistent with epithelial to mesenchymal transition, has been documented in PKD and was found by immunohistochemistry (red) in the control (no cell) group in cyst lining cells. Immunoreactive vimentin was decreased in treated (control and SAA+ cells) kidneys with most tubule lining cells positive for the epithelial marker pan-keratin (not shown). GFP+ donor cells did not stain with anti-vimentin. Kidney sections were counterstained with DAPI to stain nuclei blue. The graphs show pixel density of vimentin (x10 to facilitate visualization on one axis) and pan-keratin. #p<0.05 vs no cells, ‡ p<0.05 vs control cells

Epithelial mesenchymal transition has also been implicated in the pathogenesis of polycystic kidney disease [29] and the mesenchymal marker vimentin has been found in cystic epithelia in the PCK rat [30]. Thus, we examined the epithelial marker pan-keratin and the mesenchymal marker vimentin in donor cells and PCK kidneys. The donor cells were of epithelial origin as indicated by uniform labeling with anti-pankeratin (not shown). The majority were also positive for either OAT1 (24±3%), THP (18±3%) or AQP2 (16±2%) consistent with proximal tubule, thick ascending limb and collecting tubule phenotype, respectively [20]. Only a small proportion (<10%) stained positive for the distal convoluted tubule marker TSC. Vimentin expression was prominent in cyst lining epithelia in control PCK kidneys at study termination. This was markedly decreased in the kidneys from treated rats. GFP+ donor cells did not stain with anti-vimentin. Conversely, pan-keratin staining was significantly higher in kidneys from cell treated than from untreated rats (Fig 9).

**Fig 10:**
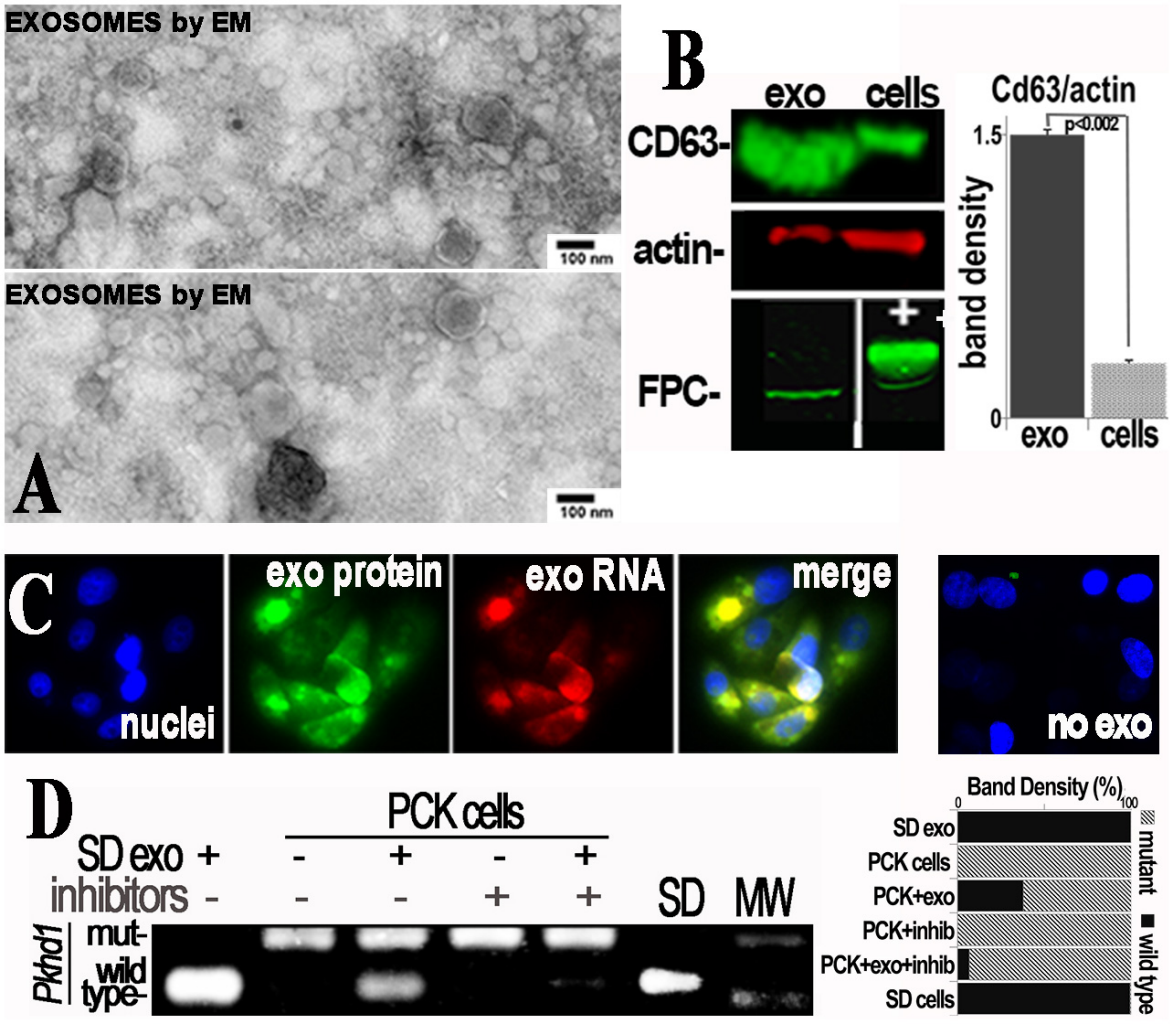
Exosome Uptake by PCK cells. Isolation of exosomes (exo) was confirmed by electron microscopy demonstrating vesicles of appropriate size after negative stain (EM, A) and enrichment of CD63 on immunoblot (B). Exosomes from normal Sprague Dawley rats express fibrocystin (FPC, B). Internal exosome protein was fluorescently labeled green and RNA red prior to incubation of PCK cells with the exosomes for 16 hours. In panel C are representative fluorescence microscopy images demonstrating uptake of the exosomes by the primary PCK renal cells. The “no exo” control was incubated with exo-glow dye but no exosomes. PCR genotyping shows expression of both wild type and mutant *Pkhd1* mRNA in the PCK cells exposed to exosomes (D). Wild type *Pkhd1* is found in SD exosomes and SD cells (SD) and only mutant *Pkhd1* in control PCK cells. After the addition of the exosome uptake inhibitor chlorpromazine and the protein synthesis inhibitor cytochalasin D prior to exosomes, no wild type *Pkhd1* was transferred to the mutant PCK cells. Graphs demonstrate quantification of band densities representing CD63 (corrected for actin) or mutant and wild type *Pkhd1*. MW, molecular weight markers, + positive control (panel B)

**Fig 11:**
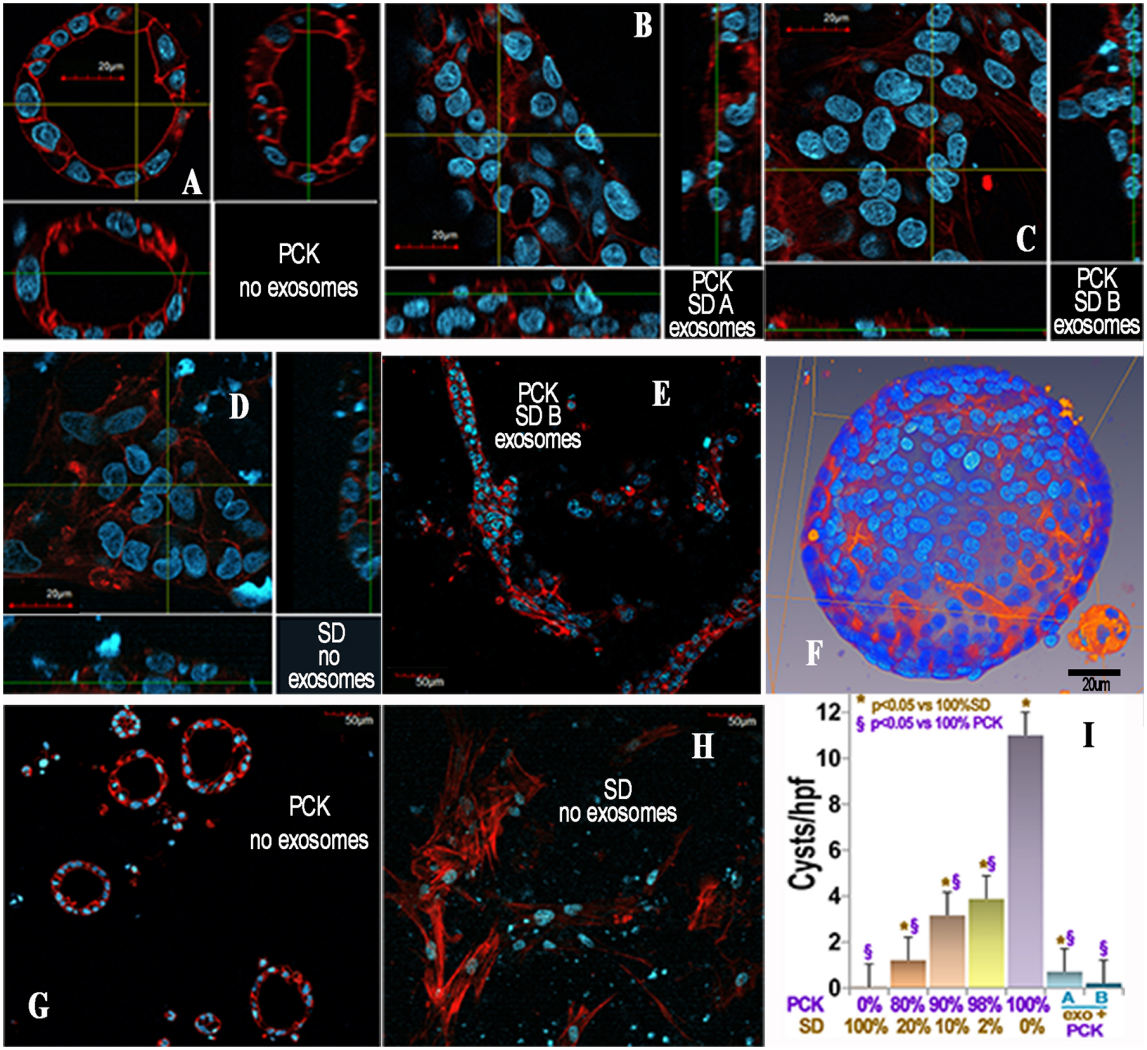
The effect of exosomes on PCK cysts in culture. Representative confocal images of cells stained with rhodamine phalloidin to label actin red and Hoescht to label nuclei blue demonstrate cysts in tubule cells from PCK rats grown in matrigel without exosome treatment (A, F, G). Neither PCK cells cultured with exosomes (B, C, E) from Sprague Dawley (SD) rats nor SD tubular cells (D, H) form cysts in matrigel. The higher power images (A-D, scale bar 20um) show orthogonal projections to demonstrate the clear presence or absence of cysts. Panel F, showing a 3D reconstruction of multiple images, also demonstrates the cystic nature of the structures formed by PCK cells (scale bar 20um). The lower power images (E, G, H, scale bar 50um) demonstrate tubular structures in the presence of exosomes, multiple cysts in one field of PCK cells and the absence of cysts in SD cells, respectively. In PCK cells treated with exosomes (E), “tube-like” structures are seen. Quantification of cyst number in a total of 168 fields is presented in the graph (I). B exosomes are from SAA+ cells, A are from SAA- control cells. Both A and B exosomes contain wild type *Pkhd1* (above).

**Fig 12:**
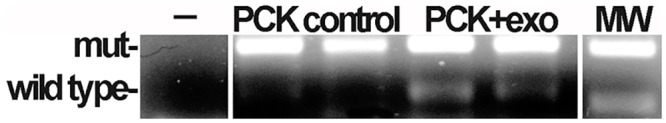
Wild type Pkhd1 in kidneys from PCK rats treated with SD exosomes. PCR genotype demonstrates mutant Pkhd1 in kidneys from control and treated PCK rats. Wild type Pkhd1 was found in kidneys from PCK rats treated with SD exosomes (24h after renal pelvic injection) but not in untreated rats. -, negative PCR control; exo, exosomes; MW, molecular weight markers.

Given that donor cells are only a small proportion of the host PCK kidney, and yet they altered the PCK phenotype, we hypothesized that engrafted cell exosomes influence neighboring PCK cells [31]. This postulate is based on the fact that exosomes contain vast mRNA libraries [32] and might carry and transfer wild type *Pkhd1* mRNA to PCK renal cells. To test this hypothesis, we verified that SD cells produce nanovesicles that express CD63 and are of a size consistent with exosomes (Fig 10) [33–35]. The SD exosomes also expressed the protein product of *Pkhd1*, fibrocystin. Intra-exosome RNA (exoRNA) and protein were labeled with Exo-Red and Exo-Green dyes (Exo-Glow, System Biosciences, Mountain View, CA), respectively. Labeled exosome cargo was taken up by cultured renal tubular cells from PCK rats, resulting in expression of wild type *Pkhd1* RNA in cells incubated with exosomes from SD cells but not in untreated PCK cells (Fig 10). When PCK cells were grown in extracellular matrix (matrigel), abundant 3D cystic structures were formed, for example Fig 11G. These cystic structures expanded for 7 days when they were imaged by 2-photon microscopy, confirming their cystic nature. SD renal cells, in contrast, did not form cysts under the same culture conditions. When PCK cells were co-cultured with renal cells derived from SD rats, the number of cysts formed decreased as the proportion of SD cells increased. When PCK cells were cultured with SD exosomes prior to incubation in matrigel, the cells remained non-cystic and formed “tubular” structures (Fig 11E). This result supports the hypothesis that exosomes derived from normal cells transfer genetic material and the presence of wild type Pkhd1 results in decreased cystogenesis in PCK cells. In addition, co-culture of PCK cells with SD cells resulted in decreased cyst formation (Fig 11). These results demonstrate that normal renal tubular cells and exosomes derived from these cells contain wild type genetic material and can improve the phenotype in polycystic kidney disease. The results are consistent with the hypothesis that improved phenotype in the presence of normal SD cells results from transfer of genetic material from the SD cells via exosomes. Injection of SD exosomes into PCK rats also resulted in the transfer of wild type *Pkhd1* mRNA into PCK kidneys (Fig 12).

## Discussion

The number of PKD end stage renal disease (ESRD) patients is increasing: US Renal Data System (USRDS) 2013 [36] reports 28.2 (per million population) PKD patients on dialysis in 1985 and 92.5 in 2011. These data illustrate that PKD is a significant clinical issue. We hypothesized that cytotherapy with cells containing the wild type *Pkhd1* gene would result in renal chimeras and improve structure and decrease cystogenesis in PKD. The PCK rat was studied because the mutated gene in the PCK rat (*Pkhd1*) is orthologous to the human gene; and the phenotype is very similar to the human phenotype in both ARPKD and autosomal dominant PKD (ADPKD) [3,37,38]. The pathophysiology of ADPKD and ARPKD, and other renal cystic diseases, is thought to result from similar abnormalities in convergent and/or integrated signaling pathways [26,38–40]. The products of the most commonly mutated genes, polycystin (PC)1 and PC2 in ADPKD and fibrocystin (FPC) in ARPKD, all localize to primary cilia and are believed to modulate essential cellular functions [41]. We report that infusion of a relatively small number of SD rat adult renal cells significantly improved the otherwise predetermined cystic phenotype of the PCK rat. Accordingly, we suggest that IRCT can be applied to limit untreatable PKD. Furthermore, multiple patients can potentially receive cells from a single donor, a critical point since many ESRD patients never receive renal transplants due to shortage of organs for donation [42]. All models have limitations, although in prior studies, we reported that donor cells expressing the tubulogenic SAA protein improved other models of CKD. However, in the PCK model, we found that the major differences existed between PCK rats receiving either SAA+ or control SAA- cells and PCK rats not receiving any cells, although those receiving SAA+ cells had slightly better renal structure (significantly decreased fibrosis) and function (significantly decreased BUN). Thus, in this model where the mechanism of renal failure is the destruction of normal renal architecture by cyst expansion, the provision of normal cells with wild type *Pkhd1* may be more critical than the role of tubulogenic SAA1.

The present results corroborate the great potential of primary renal cell transplants. In addition to containing the wild type *Pkhd1* gene, anchored donor tubular cells may positively influence recipient renal cells [20]. Thus, we have also shown that exosomes from normal SD cells contain wild type *Pkhd1* and its protein product, fibrocystin, and that wild type *Pkhd1* can be transferred from renal exosomes to PCK renal cells. In contrast to our cell transplant protocols, stem cells have not been shown to become functional renal cells [43–46] and, in some cases, the transplanted stem cells acquire a totally undesirable and uncontrolled phenotype in recipient CKD kidneys [47] or results in embolization in the lung [48]. Microvesicles from mesenchymal stem cells or endothelial progenitor cells have been shown to protect from ischemic renal injury or partial nephrectomy in rodent models [49–52]. In a model of renal failure due to ischemia followed by gentamicin, improvement in renal function and structure has been found using adult renal cells enriched for erythropoietin producing cells [53]. In the present study, the long term benefit of cell transplants on both structure and function points to a widespread impact which may be due to intra-renal delivery of the wild type *Pkhd1* gene as well as improved renal vasculature with better delivery of oxygen/nutrients. In conclusion, we suggest that, in PKD, adult renal epithelial cells and exosomes offer a novel, physiological and effective means to deliver normal genes. These potential therapies effect preservation of renal structure and function and limit cyst formation and expansion in experimental PKD.

## Supporting Information

S1 File Animal Use. This study was carried out in strict accordance with the recommendations in the Guide for the Care and Use of Laboratory Animals of the National Institutes of Health. The protocol was approved by the Institutional Animal Care and Use Committee of the Indiana University School of Medicine (permit 3616). All surgery was performed under isofluorane anesthesia and all efforts were made to minimize suffering. This included the administration of analgesia (buprenorphine) postoperatively. Although criteria (including minimal movement, not taking food, loss of more than 15% of body weight) for early euthanasia were in place, early euthanasia was not necessary. The animals were monitored regularly: continuously while under anesthesia and then daily. The method of euthanasia, overdose of a barbituric acid derivative with subsequent exsanguination, is consistent with the American Veterinary Medical Association guidelines for the Euthanasia of Animals.
(DOCX)
