# Supplementary material for: Improved Structure and Function in Autosomal Recessive Polycystic Rat Kidneys with Renal Tubular Cell Therapy
Source: PLoS One. 2015 Jul 2;10(7):e0131677. doi: 10.1371/journal.pone.0131677 (PMC4489886; doi:10.1371/journal.pone.0131677)
Supplement: S1 File — This study was carried out in strict accordance with the recommendations in the Guide for the Care and Use of Laboratory Animals of the National Institutes of Health. The protocol was approved by the Institutional Animal Care and Use Committee of the Indiana University School of Medicine (permit 3616). All surgery was performed under isofluorane anesthesia and all efforts were made to minimize suffering. This included the administration of analgesia (buprenorphine) postoperatively. Although criteria (including minimal movement, not taking food, loss of more than 15% of body weight) for early euthanasia were in place, early euthanasia was not necessary. The animals were monitored regularly: continuously while under anesthesia and then daily. The method of euthanasia, overdose of a barbituric acid derivative with subsequent exsanguination, is consistent with the American Veterinary Medical Association guidelines for the Euthanasia of Animals. (DOCX) [file pone.0131677.s001.docx]

Supporting Information—Animal Use

Housing and husbandry:

Housing (in sterile air-filtered plastic cages with paper pelleted bedding, 2 rats/cage) was in the Laboratory Animal Care Facility (LARC) of Indiana University School of Medicine, which is operated in accordance to the principles set forth in the Guide for the Care and Use of Laboratory Animals. LARC is fully accredited by the American Association for the Accreditation of Laboratory Animal Care.

The animals were maintained in 12 hour light/darkness cycles (light during day) at 70 +/- 2°F, between 30-70% relative humidity with ad-lib access to standard (Purina) rat chow and water. Species-appropriate enrichment (appropriate size cages, bedding, housing together, etc) is provided by the animal care technicians.

Animals are assessed regularly. Following surgery, incisions are checked daily until they are healed. Appetite, water consumption, general body condition and mobility are assessed daily. Analgesia is administered as needed based on the assessment. Criteria for euthanasia if the animals appeared to be suffering were in place.

Sample size:

The total number of animals was 30, dived among 6 groups. This was determined by a sample size calculation using a meaningful difference in total cyst volume of 25%, anticipated standard deviation from a collaborator (Dr. Gattone) and an on-line calculator <http://department.obg.cuhk.edu.hk/researchsupport/Sample_size_CompMeanIndependent.asp>.

Allocating animals to experimental groups:

The animals were allocated to experimental groups randomly by a blinded animal care technician. The order in which the animals in the different groups were treated and assessed was random and the researcher administering the treatments or assessing outcomes was not aware of the groups being treated or assessed.

Experimental outcomes:

The primary outcomes were cyst volume, fibrosis and genotypes. Secondary outcomes included albuminuria, blood urea nitrogen and kidney weight.

Statistical methods

The statistical methods are described in the main manuscript. The unit of analysis for animal studies was either a single animal (albuminuria, BUN) or a single kidney (as each kidney was treated differently—ischemia or sham).

Baseline data:

The animals were obtained from Charles River, Wilmington, MA, USA where they are regularly tested for viruses, bacteria and parasites and none were detected. Our LARC facility also has an extensive monitoring program and sentinel animals in place. The initial weights (202 ± 3 g) without significant differences among the groups, all were drug/test naïve at baseline.

Numbers analyzed:

All numbers were included in each analysis with the exception of the single animal that expired.

Adverse events:

none
